# European Correspondence

**Published:** 1867-12

**Authors:** W. J. Armstrong

**Affiliations:** Paris, France


### EUROPEAN CORRESPONDENCE.

PARIS, FRANCE, OCT. 25th, 1867.

*Report of a Case of Vesico-Vaginal Fistula.* Operation by DR. J. MARION SIMS. Treatment of Amputations by Exclusion of Air; Description of Apparatus; Advantages Claimed for it over the Ordinary Method.

*To Editors Atlanta Medical and Surgical Journal:*

A few days ago I witnessed the result of an operation upon a case of Vesico-Vaginal Fistula, which I had watched with much interest, both on account of the extent of the fistula, and because I was glad to be able to avail myself of the valuable experience of the operator, Dr. J. Marion Sims. As Dr. Sims is an Alabamian, many of your readers know him personally, and will, doubtless, feel interested in a brief report of the case.

The fistula was transverse, and very large, measuring nearly three inches in length. The upper part of the vesico-vaginal septum was entirely gone, no vestige of the vaginal tissue remaining attached to the cervix uteri, so that much more of the anterior part of the organ was exposed to view than it is possible to see under any other circumstances. The anterior part of the cervix also had been partly destroyed by sloughing, and the angles of the fistula were firmly bound to the inner face of the pubis on both sides by cicotrical tissue, rendering the parts immovably fixed. Of course you will agree with me in regarding this a very unfavorable case, which required more than ordinary skill to effect a cure. Dr. Sims said it was, taken all in all, one of the most difficult cases to operate on; yet he promised to cure it by one operation, and did it. The operation required a little more than an hour—a long time for him. Eleven or twelve ligatures were used, which were renewed on the ninth day.

This operation was performed in one of the hospitals here, and is the ninth hospital in which he has been invited to operate, in this city, an honor to which his superior skill and ability justly entitle him; and more just will the last remark appear, when the fact is taken into consideration, that it is only in the most difficult cases, where a cure is almost despaired of, that his services are called into requisition.

The new mode of treating amputation, by excluding air, I have seen at the l'Hotel Dieu, in the wards of M. Maisannerve, who manifests great enthusiasm, when speaking of the benefits to be gained by its adoption. Up to this time, I have seen but three cases under treatment; and, therefore, am not fully prepared to give my views on all the advantages claimed for it. I will, however, give you a description of this apparatus, and enumerate some of the advantages which this method of treating amputations is said to possess over the one usually adopted by surgeons.

It seems that M. Jules Guerin first conceived the idea that, to prevent decomposition of the fluids poured out by the stump, and secure the most favorable result, it would be necessary to exclude the air. This agent, he contended, holds certain elements in suspension, of organic origin, which cause the decomposition of the *liquor sanginis* and healthy pus, thereby interfering with the healing process, and, at the same time, endangering the life of the patient from absorption. Entertaining these views, he first applied the principle of excluding the air, by an apparatus much more complicated than the one in present use. M. Maisannerve simplified and improved the apparatus by establishing a drain, so that in addition to excluding the air there is a drain for the stump.

Inclosed you will find a rough drawing of the apparatus, taken at the bed-side, which will render its "*modus operandi*" intelligible at a glance. In the first place, then, it is composed of a cap of caoutchouc, or India rubber, in the shape of an ordinary bell jar, of convenient size to slip over the stump; leading from this cap is a tube of the same material, three feet in length, which opens through a stopper into a glass reservoir. Passing out from this reservoir, through the same stopper, is a second tube of the same length, to the end of which is attached a small air-pump. These, then, compose the different parts of the apparatus, which M. Maissannerve calls the "machine aspiration pneumatic."

When the cap is placed over the stump, and the air exhausted by the pump, the flaps are brought into perfect apposition by the atmospheric pressure from without, and, at the same time, the drain is effected by the vacuum created in the reservoir. Here, then, we have the application of two principles—the exclusion of air and a drain. Now, by means of these principles in the application of this apparatus, it is believed that many of the accidents which frequently follow amputations, such as phlegmonous erysipelas, phlebitis, pyæmia, and the burrowing of pus, will be very much lessened, if not entirely prevented. Its action, for instance, in preventing the burrowing of pus, is easily explained, when it is remembered that the pressure exerted by the atmosphere from without, prevents the flaps *bagging*, and retains them in such close apposition as to prevent its accumulation. And, in addition to this, there is the drain, which effectually removes all fluids from the stump, and discharges them in the reservoir. In the same manner is its action explained in preventing phlegmonous erysipelas, phlebitis, and pyæmia, from the absorption of decomposing animal matter. But better still will be the action of this apparatus, if it can be made to secure union, by the first intention, in all cases, as some of its advocates anticipate. The "*ultima thule*" of operative surgery will then be attained. But my observation does not lead me to believe that it will be secured in all cases. It may, perhaps, be urged, that the force exerted, or the pressure on the part, in the application of this treatment, will interfere with the circulation and nutrition of the limb. But the same reasoning applies to the application of a bandage. The force necessary to be used depends, in the one case as in the other, upon the judgment of the surgeon. The pressure upon the part is considerable, it is true; but applied equally, instead of being a disadvantage, it has certainly acted beneficially in preventing the usual amount of exudation and inflammatory action, in the cases under my observation. This, of itself, is a great desideratum, as the cure is hastened, and the suffering of the patient very much diminished. The same advantages could be claimed for this mode of treatment in wounds of the trunk. But as yet no use has been made of it, so far as I am aware, owing to the difficulty of making the application; for if it should be applied to a wound situated there, its action would be similar to that of a cupping glass in displacing the soft parts, and render its adoption impracticable. To make the apparatus available in treating a wound, the part, or limb, must be surrounded by the cap, in order to distribute the pressure equally on all sides.

W. J. ARMSTRONG.

